# Bim and PD-1 identify effector CD8 T cells responsive to anti-PD-1 therapy in metastatic melanoma patients

**DOI:** 10.1186/2051-1426-3-S2-P298

**Published:** 2015-11-04

**Authors:** Roxana Dronca, Xin Liu, Kottschade Lisa, Rob Mcwilliams, Svetomir Markovic, Haidong Dong

**Affiliations:** 1 Mayo Clinic, Rochester, MN, USA; 2 Div. of Medical Oncology, Mayo Clinic, Rochester, MN, USA; 3 Div. of Medical Oncology and Hematology, Dept. of Immunology, Mayo Clinic, Rochester, MN, USA; 4 Departments of Urology and Immunology, Mayo Clinic, Rochester, MN, USA

## 

Although PD-1 blockade therapy demonstrates promising therapeutic effects in a portion of metastatic melanoma patients, clinical outcomes remain variable, with some patients achieving durable responses, others experiencing early disease worsening followed by later tumor reduction, while some show no benefit. Thus, it is critical to identify patients with melanoma who are most likely to benefit from continued anti-PD-1 therapy, therefore increasing drug efficacy and decrease toxicity through a personalized approach to treatment. Since anti-PD-1 blockade therapy aims to block the interaction of PD-1 and its ligands (i.e. B7-H1/PD-L1), measurement of PD-1 downstream signaling molecule may enable us to monitor or predict T cell responses to anti-PD-1 therapy in melanoma patients. In this report, we established that CD8 T cells expressing high levels of Bim (a pro-apoptosis molecule) and PD-1 are effector cells that are responsive to anti-PD-1 therapy. Among tumor-related CD11a high CD8 T cells in the peripheral blood of metastatic melanoma patients, Bim expression was significantly associated with expressions of PD-1 and effector cell markers (T-bet and granzym B). In human tumor tissues, Bim expression was strongly correlated with B7-H1 and PD-1 expression. Genetic absence of PD-1 prevented Bim up-regulation in tumor-related CD8 T cells in tumor tissues. B7-H1 fusion protein or B7-H1-expssred by tumor cells directly induced Bim protein up-regulation in pre-activated CD8 T cells via regulation of AKT activation and phosphorylation of Bim. Bim up-relation was coincident with T cell apoptosis induced by B7-H1. In metastatic melanoma patients, the frequency of Bim+ PD-1+ CD8 T cells significantly increased in the peripheral blood compared to healthy donors. Importantly, the baseline frequency of Bim+ per PD-1+ CD8 T cells in the peripheral blood of melanoma patients was higher in responders to anti-PD-1 therapy than in non-responders. After four cycles of anti-PD-1 treatment, the frequency of Bim+ per PD-1+ CD8 T cells dramatically decreased in the peripheral blood of responders than in non-responders. Our results indicate that Bim is a downstream signaling molecule of PD-1, which reflects the degree of PD-1 interaction with its ligand (B7-H1) in effector CD8 T cells. Measurement of the frequency of Bim in tumor-related PD-1+ CD8 T cells in the peripheral blood of metastatic melanoma patients may provide a less invasive method to monitor or predict responses to anti-PD-1 therapy.

